# Therapeutic decisions under uncertainty for spinal muscular atrophy: The DECISIONS-SMA study protocol

**DOI:** 10.1371/journal.pone.0264006

**Published:** 2022-02-15

**Authors:** Gustavo Saposnik, Paola Díaz-Abós, Victoria Sánchez-Menéndez, Carmen Álvarez, María Terzaghi, Jorge Maurino, María Brañas-Pampillón, Ignacio Málaga

**Affiliations:** 1 NeuroEconSolutions (Neuroeconsolutions.com), Toronto, Canada; 2 Clinical Outcomes & Decision Neuroscience Unit, Li Ka Shing Institute, University of Toronto, Toronto, ON, Canada; 3 Division of Neurology, Department of Medicine, St. Michael’s Hospital, University of Toronto, Toronto, ON, Canada; 4 Medical Department, Roche Farma, Madrid, Spain; 5 Department of Pediatric Neurology, Hospital Universitario Central de Asturias, Oviedo, Spain; Providence Care Hospital, CANADA

## Abstract

**Background:**

The therapeutic landscape for spinal muscular atrophy has changed in the last few years, encompassing respiratory/motor function and life expectancy benefits. However, physicians still have the challenge of tailoring individuals’ treatment to therapeutic goals, disease progression, patient/caregiver’s preferences, and personal experience to achieve an optimal risk/benefit balance. This study aims to provide insight into the preferred treatment choices of pediatric neurologists managing spinal muscular atrophy in their daily practice and to recognize behavioral factors that may influence decision-making.

**Methods:**

This is a noninterventional, cross-sectional pilot study involving 50 pediatric neurologists managing spinal muscular atrophy in Spain. We designed an online platform that contains 13 simulated case scenarios of common presentations of patients with spinal muscular atrophy. The primary study outcome will be treatment preferences according to the percentages of participants who select treatment initiation when recommended, switch therapies when there is evidence of disease progression, and select treatment discontinuation when disease progression puts patients outside treatment recommendation (11 case scenarios). Secondary outcomes include therapeutic inertia prevalence (11 case scenarios), herding phenomenon prevalence (2 case scenarios), care-related regret prevalence (specific questions) and intensity (10-item Regret Intensity Scale), occupational burnout prevalence (nonproprietary single-item measure), and risk preferences (uncertainty test and risk aversion assessment).

**Conclusions:**

The study findings will contribute to better understand relevant factors associated with therapeutic decisions of pediatric neurologists in spinal muscular atrophy, identifying treatment preferences and evaluating the role of behavioral aspects such as therapeutic inertia, herding, regret, and workplace burnout.

## Introduction

**Fig 1:**
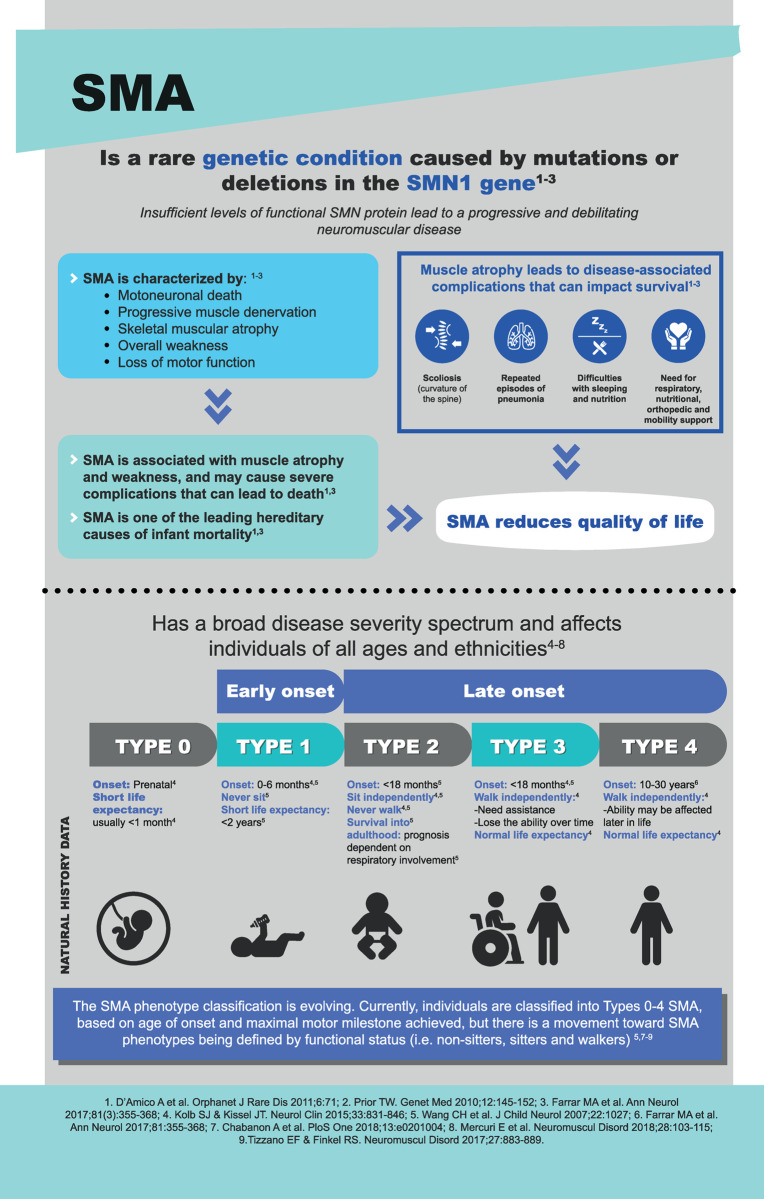
Spinal muscular atrophy outline and impact.

Spinal muscular atrophy (SMA) is an autosomal recessive neuromuscular disease caused by homozygous deletion or mutation of the survival motor neuron 1 gene on chromosome 5q13 that leads to progressive muscle weakness and atrophy [1–3]. SMA is categorized into clinical subtypes based on the age at onset and severity of symptoms [1–3], mainly affecting infants (types I and II) and children (type III) [1–4]. The disease causes a wide range of clinical symptoms, including respiratory, nutritional, orthopedic, rehabilitative, emotional, and social disorders [1,3,5], which may seriously compromise patients’ health and cause a considerable impact on the health-related quality of life of both patients and their caregivers [6–8] (Fig 1).

**Fig 2:**
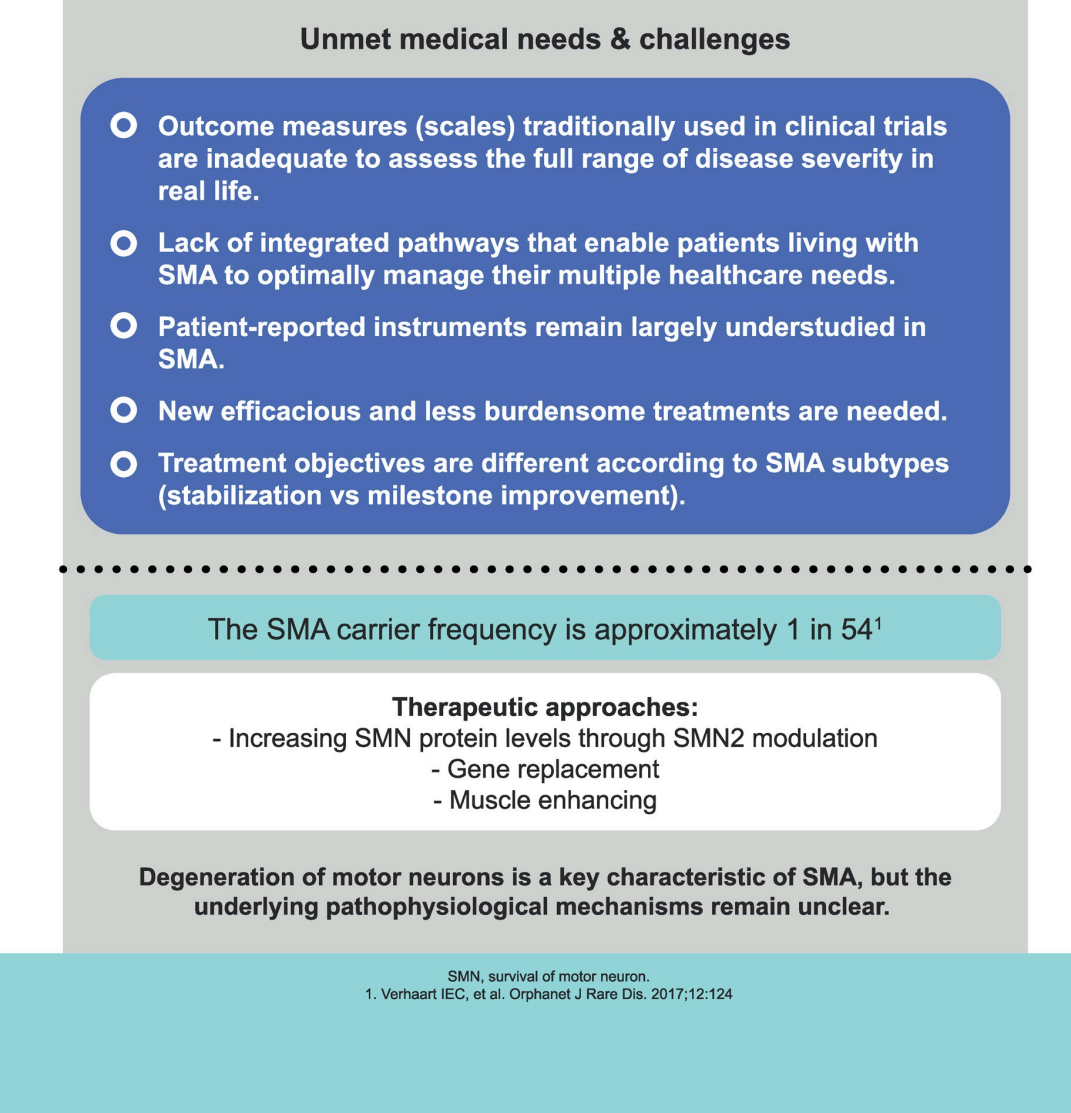
Medical needs and challenges for spinal muscular atrophy.

The SMA therapeutic landscape has changed over the last few years with the appearance of different therapeutic approaches such as antisense oligonucleotides, small molecules, or gene therapy [1,4,9,10]. The administration of these therapies made it possible for SMA patients’ respiratory and motor function to be stabilized or even improved, as well as increasing their life expectancy [4,9]. However, physicians still have the challenge of tailoring each individual’s treatment according to therapeutic goals, disease progression, patients’ and caregivers’ preferences, and their personal experience to achieve an optimal risk/benefit balance [11–13] (Fig 2).

Making this complex decision involves educating healthcare professionals and parents on the disease’s course and complications [14]. Despite the limited evidence-based understanding of how physicians make treatment decisions when managing SMA, therapeutic options seem to be usually assessed according to their clinical experience when exposed to the uncertainties of new agents [14]. However, decision making may also be influenced by cognitive or behavioral biases [15,16], including personality traits and background effects such as overconfidence, uncertainty tolerance, anchoring effect, information availability, or confirmation biases [17]. Behavioral economics is the science that studies the principles of how we make decisions, combining psychology and economics to comprehensively understand cognitive and behavioral biases [15]. It can therefore contribute to clarifying how physicians make their decisions and translate this into policy interventions that ultimately improve patients’ healthcare [15].

Considering the above, this study aims to provide insight into therapeutic decision-making for SMA using behavioral economics paradigms, identifying treatment preferences of pediatric neurologists routinely managing SMA and recognizing the role of behavioral factors such as therapeutic inertia, herding phenomenon, care-related regret, occupational burnout, and risk preferences.

## Materials and methods

### Study design and participants

**Fig 3:**
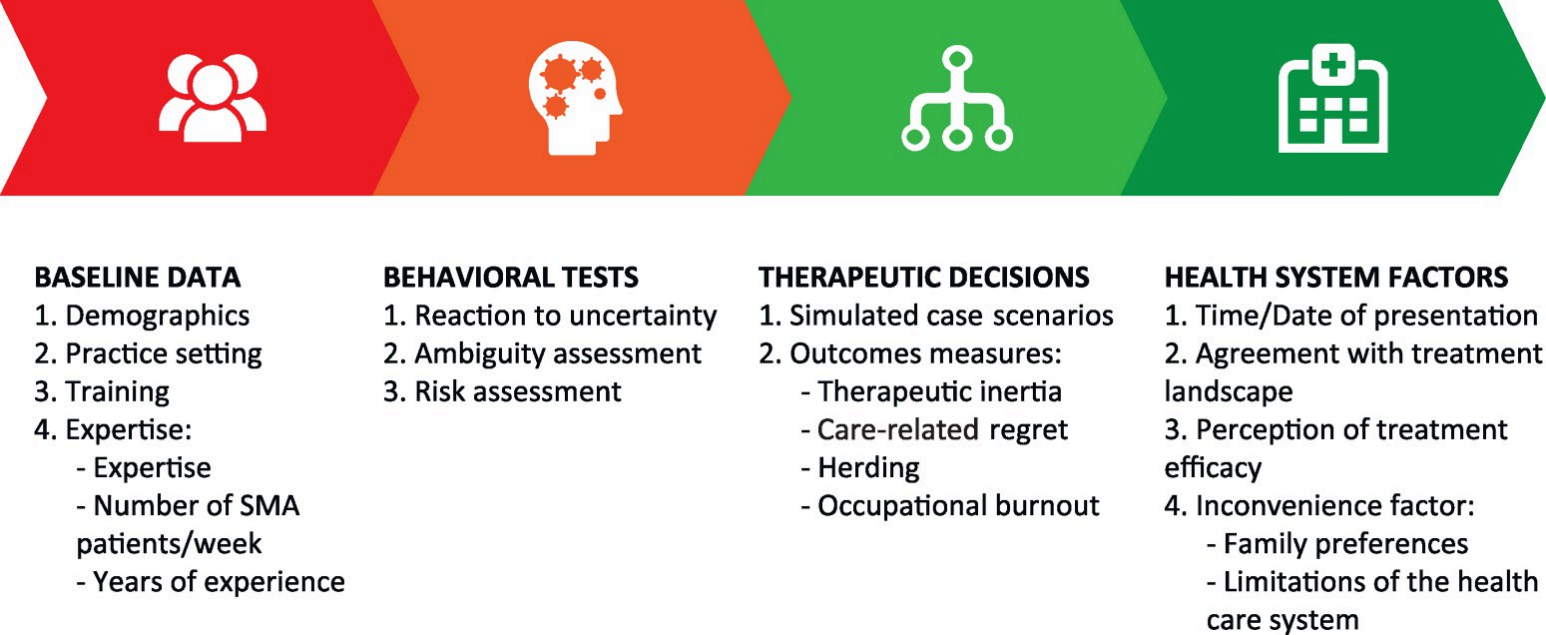
Study flow.

This is a noninterventional, cross-sectional, web-based pilot study involving 50 pediatric neurologists with expertise in managing patients with SMA in their routine clinical practice in Spain. Pediatric neurologists will be invited to participate by the Spanish Society of Pediatric Neurology (SENEP). The selection criteria also include participants practicing in academic or nonacademic settings, general practice pediatric neurologists or those specialized in neuromuscular disorders, involved or not in clinical research, from across Spain (Fig 3).

### Study objectives

The primary study objective is to assess pediatric neurologists’ treatment preference for SMA in terms of its initiation, switch, and discontinuation.

Secondary study objectives include evaluating therapeutic inertia, herding phenomenon, care-related regret, occupational burnout, and risk preferences of pediatric neurologists routinely managing SMA.

### Outcome measures and definitions

#### Treatment preferences

The pediatric neurologists’ treatment preferences will be assessed according to their choices in eleven simulated case scenarios (S1 Supporting information). Case-scenarios were originally designed by our research team (GS, PDA, JM, MBP, and IM) derived from the most common situations experienced by SMA patients in clinical practice and reviewing clinical trials and patient/caregivers preferences literature [9–11]. The study (simulated case scenarios, questionnaires and scales) will be conducted in Spanish.

The primary outcome variable will be pediatric neurologists’ treatment preference according to: 1) the percentage of participants who select treatment initiation when recommended [18–22], 2) the percentage of participants who select treatment switch when there is evidence of disease progression (i.e., a decrease in baseline scale score greater than the scale’s minimal clinically important difference) with initial therapies [23,24], and 3) the percentage of participants who select treatment discontinuation when disease progression puts patients outside treatment recommendation [18–22].

#### Therapeutic inertia

Therapeutic inertia is defined as the absence of treatment initiation or intensification when treatment goals are unmet [25]. The study outcome measure will be its prevalence according to the pediatric neurologist responses on eleven case scenarios designed ad hoc (S1 Appendix). Its presence will be identified according to a score defined as the number of case scenarios that fit therapeutic inertia over the total number of presented cases [16]. This score may therefore range from 0 to 11. Participants with a score of ≥1 (i.e., therapeutic inertia in at least one case scenario) will be considered to calculate therapeutic inertia prevalence.

#### Herding phenomenon

Herding is a phenomenon by which individuals follow others’ behavior rather than deciding independently based on their own private information [26]. It has been shown that herding may lead to suboptimal decisions [26,27]. The prevalence of herding will be assessed using two case scenarios designed ad-hoc (S1 Appendix). Its presence will be identified when the participant’s responses denote herding in at least one case scenario.

#### Care-related regret

Regret is an emotion experienced when one believes that the current situation would have had a better outcome by choosing a different course of action [28]. Care-related regret was associated with suboptimal choices by healthcare professionals [29]. Specific questions will assess the presence of regret, and its intensity will be evaluated using the 10-item Regret Intensity Scale (RIS-10). The specific questions will determine the presence of regret related to any patient and SMA patient situation within the last 5 years. The RIS-10 is a validated tool to assess care-related regret caused by a past event, covering affective, physical, and cognitive aspects [30]. For each item, participants will be asked to rate their agreement on "how they feel now" from 1 (strongly disagree) to 5 (strongly agree). The RIS-10 overall score may range from 1 to 5, with higher scores indicating higher regret intensity.

#### Occupational burnout

Burnout is a condition characterized by emotional exhaustion, depersonalization, and a low sense of personal accomplishment [31]. Physicians’ burnout is a common phenomenon which may influence therapeutic decisions [32–34]. The prevalence of occupational burnout among participating pediatric neurologists will be calculated according to their scores on a nonproprietary single-item burnout measure, which instructs respondents to rate their burnout level based on their own definition of burnout on a 5-point scale [31]. The absence or presence of burnout will be dichotomized according to the following scores: ≤2 (no symptoms of burnout) versus ≥3 (1 or more symptoms) [31].

#### Risk preferences and tolerance to uncertainty

Physicians’ low tolerance to uncertainty has been associated with suboptimal decisions and therapeutic inertia [35]. Tolerance to uncertainty will be assessed using the standardized physician’s reaction to an uncertainty test [36,37]. A short version following a factor analysis comprises five questions showing reliable psychometric properties [35,38,39]. Participants will rate their level of agreement with each question from 0 (strongly disagree) to 5 (strongly agree), and a total score will be calculated [38]. Low tolerance to uncertainty will be defined as values below the median of the total score [35].

Risk aversion, defined as the tendency to prefer safe payoffs over probabilistic payoffs when the expected value is kept constant [40,41], will also be assessed. A risk-averse participant would prefer a treatment that provides a slight improvement with certainty over a therapy that offers a larger or no improvement with equal chance (50/50). We will evaluate risk aversion by identifying the safe amount for which a participant is indifferent between the safe and the risky option [42]. Participants will be asked about the minimal amount of money they would prefer instead of the equiprobable gamble of winning €400 or €0 (expected value of €200). The degree of risk aversion of each individual will correspond to the difference of the expected value of the risky option (€200) minus the participant’s response (proxy of certainty equivalent) [35].

### Data management

The data source will be the pediatric neurologists participating in the online study. Their data will be recorded in a database specifically designed for this research project through an electronic case report form. Pediatric neurologists will electronically give their written informed consent, confirm their eligibility, and provide some information about their profile (e.g., age, gender, academic/research profile, years of experience). They will then be presented with several case scenarios and scales to capture their feedback and opinion on study outcomes.

After recording all the data from the last pediatric neurologist participating in the study and resolving any potential inconsistency, the study database will be locked, and the statistical analyses will be performed.

### Statistical considerations

According to the Spanish Society of Pediatric Neurology, there are over 90 pediatric neurologists and 35 neuromuscular hospital-based clinics in Spain. Our previous experience in decision-making studies performed in Spain supports a response rate higher than 50% [35]. This exploratory pilot study’s sample size is estimated at 50 participants, given the limited number of pediatric neurologists managing SMA patients in Spain.

The study outcomes will be analyzed descriptively, calculating frequency distributions of qualitative variables, measures of central tendency and dispersion of quantitative variables, and 95% confidence intervals. Regression models will also be built for primary and secondary outcome measures to adjust their results for participant characteristics.

Only available data will be considered in the analyses. Unavailable data will be described as missing, without any imputation/allocation. The statistical analysis will be performed using Stata Statistical Software 13.0 (StataCorp., College Station, TX, USA) and considering a significant level of 0.05.

### Ethical considerations

This study will be conducted according to the Guidelines for Good Pharmacoepidemiological Practice published by the International Society of Pharmacoepidemiology, the ethical principles laid down in the World Medical Association Declaration of Helsinki, and applicable national regulations. The study was approved by the ethics committee of Hospital Clínico San Carlos (Madrid, Spain), and all participants will give their written informed consent before collecting any study data.

### Study status and timeline

The study status is ongoing. Participant recruitment and data collection are planned to begin in June 2021. The expected date for database completion is December 2021.

## Discussion

This noninterventional pilot study will contribute to better understand the therapeutic decision-making process of pediatric neurologists who routinely care for SMA patients in Spain. The uniqueness of this study is that it uses a behavioral paradigm approach to examine the role of herding phenomenon, care-related regret, occupational burnout, and risk preferences in therapeutic decisions related to SMA (Figs 1 and 2). Our study will assess treatment preferences and factors associated with therapeutic inertia among pediatric neurologists.

The change in the SMA treatment landscape that has taken place in the past few years has increased the therapeutic possibilities and decisions made by SMA patients, their caregivers, and healthcare providers. The assessment of patients and caregivers’ treatment-related priorities, expectations, and risk weighting for decision making has since gained relevance [43–46]. However, the information available on how current therapeutic choices are made from pediatric neurologists’ perspective is still lacking.

A recently published survey aimed to improve understanding of SMA patients and caregivers’ treatment choices, considering that their health status and life experience may influence how they perceive changes concerning desired benefits or therapeutic risks [44]. Similarly, clinical neurologists’ experiences managing SMA in their daily practice may affect their perception of disease-related changes and risk preferences. Indeed, experiencing the exhaustion derived from occupational burnout can translate into emotional distress and decreasing engagement which may affect physician decisions and patient outcomes [31]. Physicians’ care-related regret was also reported to negatively impact their health, quality of life, and patient care, as well as leading physicians to talk more often to their colleagues in order to improve their clinical practices [30]. Although group support may play an important role in enhancing clinical practices, it may also lead physicians to follow therapeutic recommendations that are not supported by best practice guidelines. This herding-like behavior has been reported as a frequent phenomenon among neurologists managing other conditions such as multiple sclerosis, with a higher occurrence under uncertainty and leading to suboptimal decisions [26]. In this scenario, therapeutic inertia could partly explain the neurologist’s resistance to escalate patient therapies under uncertainty, such as controversial situations or unclear efficacy evidence [25,35]. Neurologists’ risk profile may therefore affect how they face decision making in these uncertain situations, with more therapeutic inertia among those showing strong aversion to ambiguity and low tolerance of uncertainty [35]. Taken together, our results will inform about educational interventions in medical education to overcome knowledge-to-action gaps in the new therapeutic landscape of SMA.

Here we describe a noninterventional study that will assess pediatric neurologists’ preferences for SMA treatment and behavioral factors that may affect their decisions using hypothetical case scenarios and specific scales/questions. This study will therefore contribute to expanding our evidence-based understanding of therapeutic decision-making for SMA.

The authors acknowledge study limitations that should be considered, such as its exploratory pilot nature. Although pediatric neurologists managing SMA in their daily practice will be invited from all around Spain, we cannot exclude the possibility of sample biases derived from their final decision to participate. We should also keep in mind that the study hypothetical case scenarios show the most common situations faced in routine clinical practice, but they do not cover the whole case mix of the disease. In addition, we cannot rule out the possibility of residual confounders, despite the comprehensive adjustments that will be performed in the analyses. Therefore, further research would be desirable to confirm the study findings and explore their generalizability to other countries with different backgrounds and healthcare systems.

In conclusion, this study will provide valuable insights into the treatment preferences of pediatric neurologists managing SMA in their daily practice, which is especially important considering the growing relevance of clinical decision-making based on values in the current healthcare system, the increasing possibilities of therapeutic approaches for SMA, and the lack of studies focusing on this subject. Following a behavioral paradigm for this assessment, this study aims to cover additional knowledge gaps in areas such as therapeutic inertia, herding phenomenon, care-related regret, occupational burnout, and risk preferences, which may also affect pediatric neurologists’ decision-making. These data will provide meaningful evidence to understand decision making when managing SMA in routine clinical practice.

## Supporting information

S1 Appendix Case scenarios as presented to participants. (DOCX)
