# Supplementary material for: Therapeutic decisions under uncertainty for spinal muscular atrophy: The DECISIONS-SMA study protocol
Source: PLoS One. 2022 Feb 15;17(2):e0264006. doi: 10.1371/journal.pone.0264006 (PMC8846509; doi:10.1371/journal.pone.0264006)
Supplement: S1 Appendix — (DOCX) [file pone.0264006.s001.docx]

**S1 Appendix. Hypothetical case scenarios.**

***Patient profiles***

1. A 5-month-old patient with recently diagnosed type 1. SMA Still does not sit. You observe mild suction and swallowing difficulties. CHOP INTEND=28.

What we would like to know about this patient is if the introduction of the new treatments will change therapeutic inertia, if with these new treatments the neuropediatrician will consider the other options as the first option, even if there is less experience with them, and perhaps it would be interesting to see, if possible, the perspective on efficacy of all the treatments.

1. On a scale from 1% to 100%, what expectation of improvement do you have with the current treatments at 2 years?
2. Which of the following options would you choose? CHOOSE 1 OPTION ONLY

- Intrathecal drug with loading dose and subsequent administration every 4 months.
- Oral drug with daily administration.
- Intravenous drug with single administration.
- **Start combined treatment with sodium valproate and L-carnitine**.
- **Do not initiate new treatment, start rehabilitation program and reassess patient in 6 months**.

1. Type 1 patient on treatment with nusinersen since 6 months of age with controlled motor milestones. The patient is currently 18 months old and has started to walk (CHOP INTEND: 54; HMFSE: 48), but has swallowing difficulties and is starting to have a wet voice.

What we would like to know about this patient is if physicians grant sufficient importance to the aspects most affecting patients’ quality of life and if they consider switching to another treatment only taking into account these variables affecting patients other that motor function, or if, for example, due to therapeutic inertia, they maintain this treatment and perform reassessment.

1. Which of the following options would you choose? CHOOSE 1 OPTION ONLY

- **Continue current treatment.**
- Discontinue current treatment and start oral drug with daily administration.
- **Continue current treatment and reinforce with speech therapist (including respiratory rehabilitation) to improve patient's bulbar functions**.
- Discontinue current treatment and start a drug with a different mechanism of action that acts on the SMN1 gene.
- **Continue current treatment and reinforce with speech therapist (including respiratory rehabilitation) to improve patient's bulbar functions**.

1. Type 1 patient treated with nusinersen since 6 months of age who at 2 years of treatment has not achieved the expected motor milestones (according to protocol of Spanish Ministry of Health).

What we would like to know about this patient profile is if when the neuropediatrician stops administering Spinraza per protocol, he/she considers using a treatment with a similar MoA because motor milestones are the priority, or prefers to leave the patient with no alternative treatment.

1. Which of the following options would you choose? CHOOSE 1 OPTION ONLY

- **Continue current treatment.**
- Discontinue current treatment and start oral drug with daily administration.
- Discontinue current treatment and start gene therapy.
- **Continue current treatment and** **reinforce with rehabilitation therapy with virtual reality to improve motor function**.
- **Discontinue current treatment and reinforce with rehabilitation therapy with virtual reality to improve motor function**.

1. A 3-year-old type 1 patient who has received treatment with onasemnogene abeparvovec since the onset of symptoms. In the last 2 months has begun to have difficulty to run and climb stairs. The patient's HINE score has decreased 4 points versus the baseline assessment.

What we would like to know about this patient profile is if the neuropediatrician will treat the patient initially with Zolgensma (we could consider this less conservative) and if the therapy fails consider concomitant treatment with other alternatives or what is the maximum time given for Zolgensma to be effective.

1. Which of the following options would you choose? CHOOSE 1 OPTION ONLY

- Start oral treatment with daily administration.
- **Intensify motor rehabilitation program as the main treatment line**.
- Start intrathecal treatment with administration every 4 months.
- **Do not initiate new treatment, start rehabilitation program and reassess patient in 6 months**.
- **Start combined treatment with sodium valproate and L-carnitine**.

***Type 2 case profiles***

1. A 4-year-old type 2 patient who has remained stable without treatment but with decreased independent for activities of daily living and increased fatigue.

What we would like to know about this patient profile is the importance given by neurologists to milestones other than motor milestones, if they have a conservative attitude towards naïve patients or if they see any worsening do they perform any treatment intervention.

1. Which of the following options would you choose? CHOOSE 1 OPTION ONLY

- Intrathecal drug with loading dose and subsequent administration every 4 months.
- Oral drug with daily administration.
- Intravenous drug with single administration.
- **Intensify motor rehabilitation treatment to maintain/improve patient's daily functions (without drug)**.
- **Start emotional support and motor rehabilitation to maintain/improve patient's daily functions (without drug)**.

1. A 1-year-old patient recently diagnosed with type 2 SMA. You discuss treatment options with the parents. The parents have no preference regarding the form of treatment administration.

What we would like to know for this patient profile is if the patient starts have more advanced motor worsening of the disease, if it is treated rapidly and if, depending on the profile, a more convenient therapy is applied even if there is less experience.

1. On a scale from 1% to 100%, what expectation of improvement do you have with the current treatments at 2 years?
2. Which of the following options would you choose? CHOOSE 1 OPTION ONLY

- Intrathecal drug with loading dose and subsequent administration every 4 months.
- Oral drug with daily administration.
- Intravenous drug with single administration.
- **Intensify motor, respiratory and swallowing rehabilitation treatment to maintain/improve patient's daily functions (without drug)**.
- Combine gene therapy with oral drug.
- Combine gene therapy with intrathecal drug.

1. A 6-year-old type 2 patient treated with nusinersen with a decrease in motor milestones at 2 years of treatment and a 3-point reduction in the HMFSE (current 58) versus baseline.

What we would like to know in this patient profile is if the patient has not yet responded to Spinraza, they have a conservative attitude and wait to see how the patient responds over time or instead decide to administer another therapy with a similar MoA to see if it works.

1. Which of the following options would you choose? CHOOSE 1 OPTION ONLY

- **Continue current treatment.**
- Discontinue current treatment and start oral treatment.
- **Continue current treatment and rehabilitation and closely monitor the patient**.
- Discontinue current treatment and treatment with gene therapy.
- **Discontinue current treatment and check on patient's progress in 3 months**.

1. A 5-year-old type 2 patient treated with nusinersen for 2 years with slight worsening of motor function and a 1-point reduction in the HMFSE (baseline 50, current 49).

What we would like to know for this patient profile is the importance granted to motor milestones when a clinically relevant change, such as a substantial improvement on the scales, is not observed, and if, depending on this, treatment with Spinraza is continued or not.

1. Which of the following options would you choose? CHOOSE 1 OPTION ONLY

- **Continue current treatment and monitoring.**
- Discontinue current treatment and start oral treatment.
- **Continue current treatment and rehabilitation and closely monitor the patient**.
- Discontinue current treatment and start gene therapy.
- **Discontinue current treatment and check on patient's progress in 3 months**.

1. A 2-year-old type 2 stable patient treated with nusinersen for 2 years who had a 6-point improvement in the HMFSE (current 58). The parents report that the child is beginning to have difficulties eating by himself.

What we would like to know about this patient profile is if, though criteria to continue treatment are met, the neurologist considers switching for aspects related to the patient's quality of life, even if motor milestones are maintained or improved.

1. Which of the following options would you choose? CHOOSE 1 OPTION ONLY

- **Continue current treatment.**
- Discontinue current treatment and start oral treatment.
- **Continue current treatment and rehabilitation and closely monitor the patient**.
- Discontinue current treatment and treatment with gene therapy.
- **Discontinue current treatment and check on patient's progress at three months**.

1. A 16-year-old type 2 patient, first seen in Spain who came without treatment and a history of late diagnosis, currently in wheel chair and with limited mobility in upper limbs.

What we would like to know about this patient profile is if in a naïve patient who arrives in an advanced phase due to lack of treatment, the neuropediatrician considers starting treatment in this phase, what drug class and what drug would be used to initiate treatment and, if possible, the perspective on efficacy in these patients.

1. On a scale from 1% to 100%, what expectation of improvement do you have with the current treatments at 2 years?
2. Which of the following options would you choose? CHOOSE 1 OPTION ONLY

- Intrathecal drug with loading dose and subsequent administration every 4 months.
- Oral drug with daily administration.
- Intravenous drug with single administration.
- **Do not initiate new treatment, start rehabilitation program and reassess patient in 6 months**.

1. A 15-year-old type 2 ambulant patient diagnosis at 3 years of age. No prior treatment received and currently stable with regard to motor milestones in the last 2 years.

What we would like to know about this patient profile is if in a type 3 naïve patient who remain ambulant and stable in motor milestones without prior treatment, the neuropediatrician considers starting treatment in this phase, what drug class and what drug would be used to initiate treatment and, if possible, the perspective on efficacy in these patients.

1. On a scale from 1% to 100%, what expectation of improvement do you have with the current treatments at 2 years?
2. What drug would you choose as first treatment option if all were available?

- Intrathecal drug with loading dose and subsequent administration every 4 months.
- Oral drug with daily administration.
- Intravenous drug with single administration.
- **Do not initiate new treatment, start rehabilitation program and reassess patient in 6 months**.

***Herding cases profile***

1. A 15-month-old patient with type 1 DMA treated with nusinersen since 8 months of age and clinically stable (CHOP INTEND=54). The parents consulted a UK university professor expert in SMA with the goal of optimizing the treatment, who suggesting switching treatment to gene therapy. You assess the child in your clinic and after talking with the parents decide on one of the following options:

- Continue current treatment as the patients has worsened clinically.
- **Discontinue current treatment and start gene therapy as suggested in the UK**.
- Discontinue current treatment and continue with rehabilitation only.
- Discontinue current treatment and start oral treatment.

1. A 9-month-old patient with type 1 SMA (CHOP INTEND=34). The family consults a neuropediatrician from another hospital who suggest that drug therapy is not warranted by the risk/benefit balance. The neuropediatrician suggests rehabilitation and speech therapy. The family consults you to know your opinion. Which of the following options would you recommend?

- Start treatment with intrathecal therapy.
- Start treatment with gene therapy.
- Start oral treatment.
- **Start rehabilitation, speech therapy and close clinical monitoring as followed in the other hospital**.
